# Supplementary figures and images for: Assembly of neuron- and radial glial-cell-derived extracellular matrix molecules promotes radial migration of developing cortical neurons
Source: eLife. 2024 Mar 21;12:RP92342. doi: 10.7554/eLife.92342 (PMC10957175; doi:10.7554/eLife.92342)

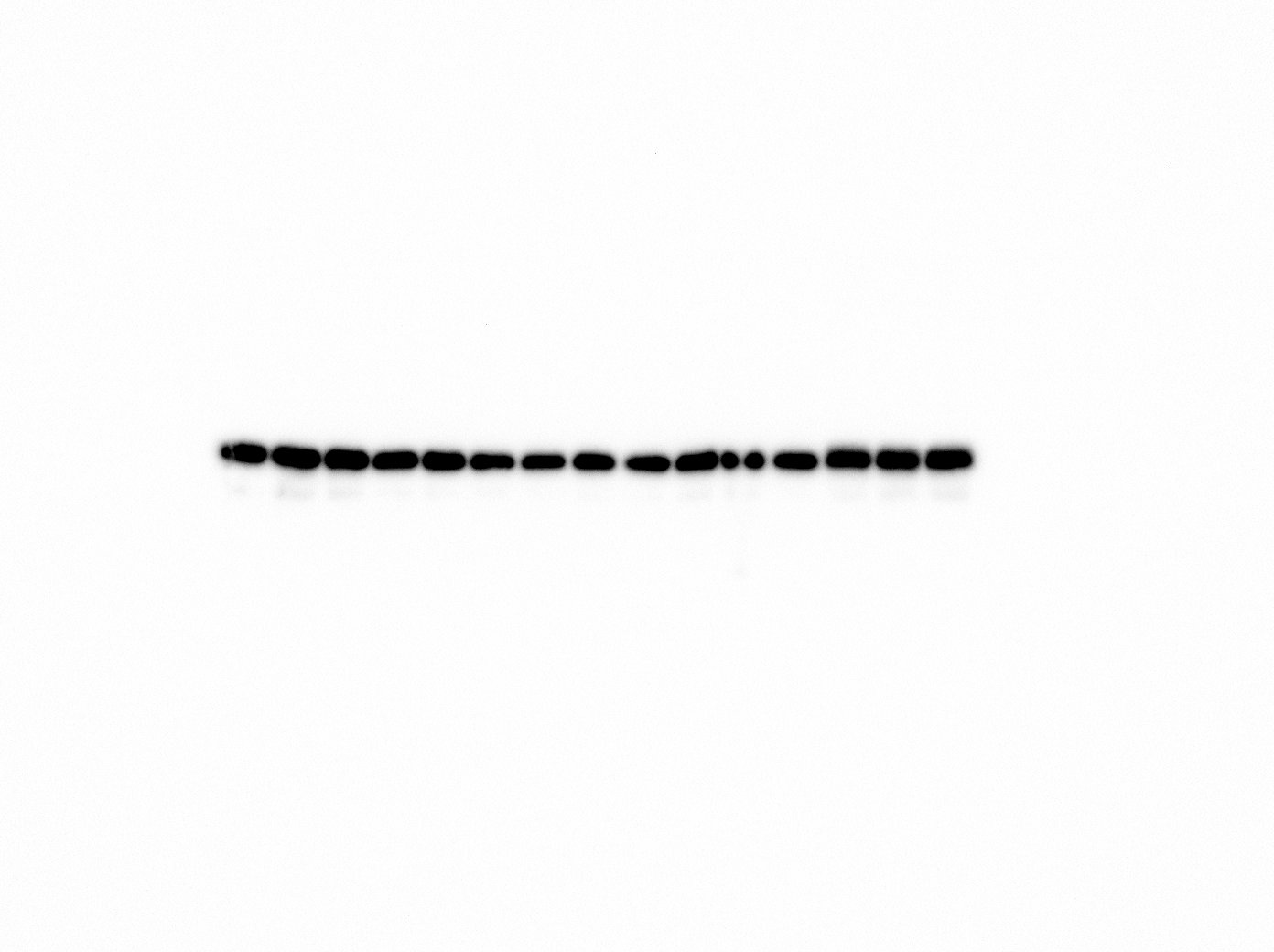

Supplement: Figure 1—source data 1. [file elife-92342-fig1-data1.zip › Figure 1-source data/Original blots for Fig1e/Fig1e GAPDH (1).jpg]

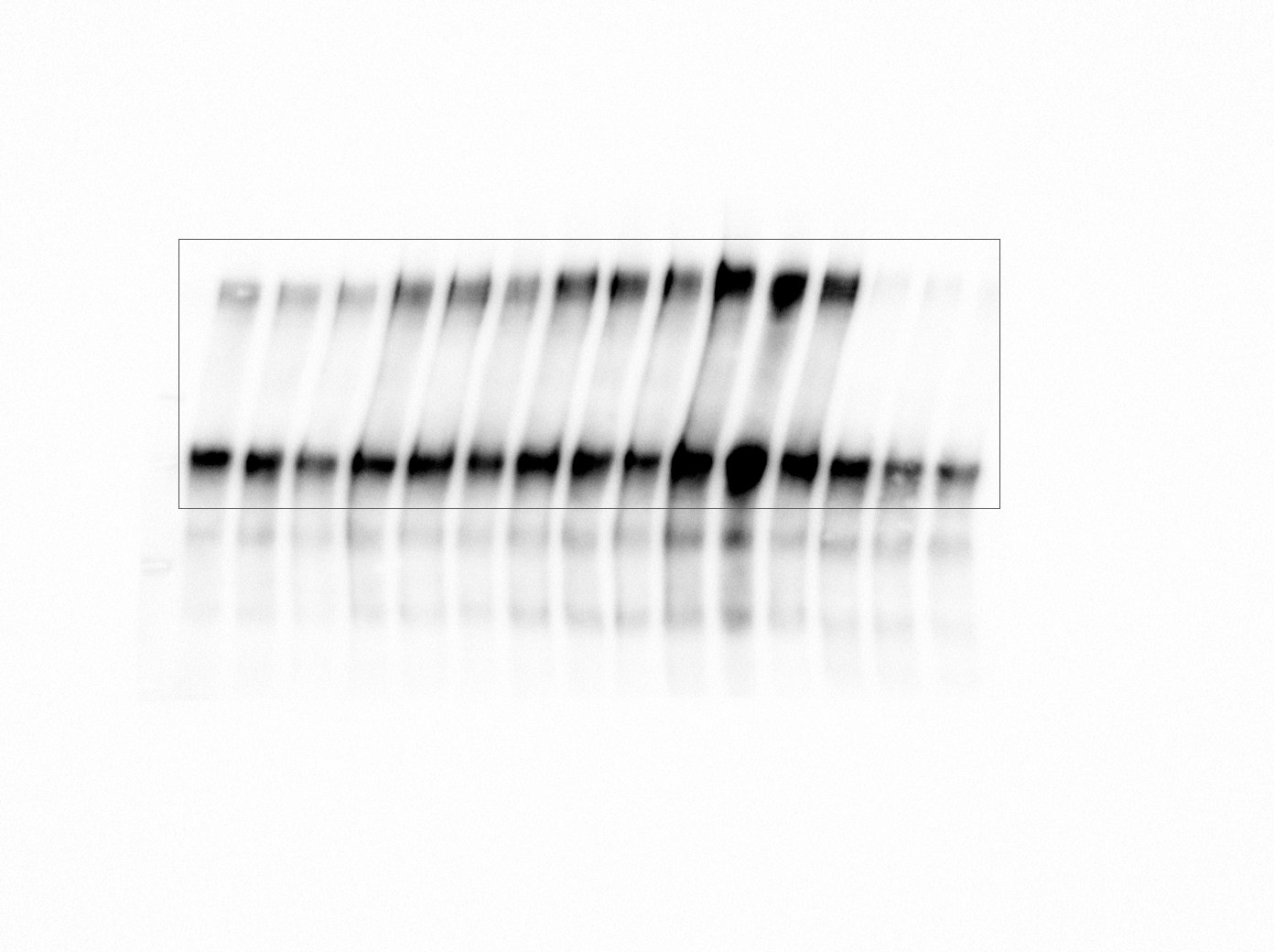

Supplement: Figure 1—source data 1. [file elife-92342-fig1-data1.zip › Figure 1-source data/Original blots for Fig1e/Fig1e NCAN (2).jpg]

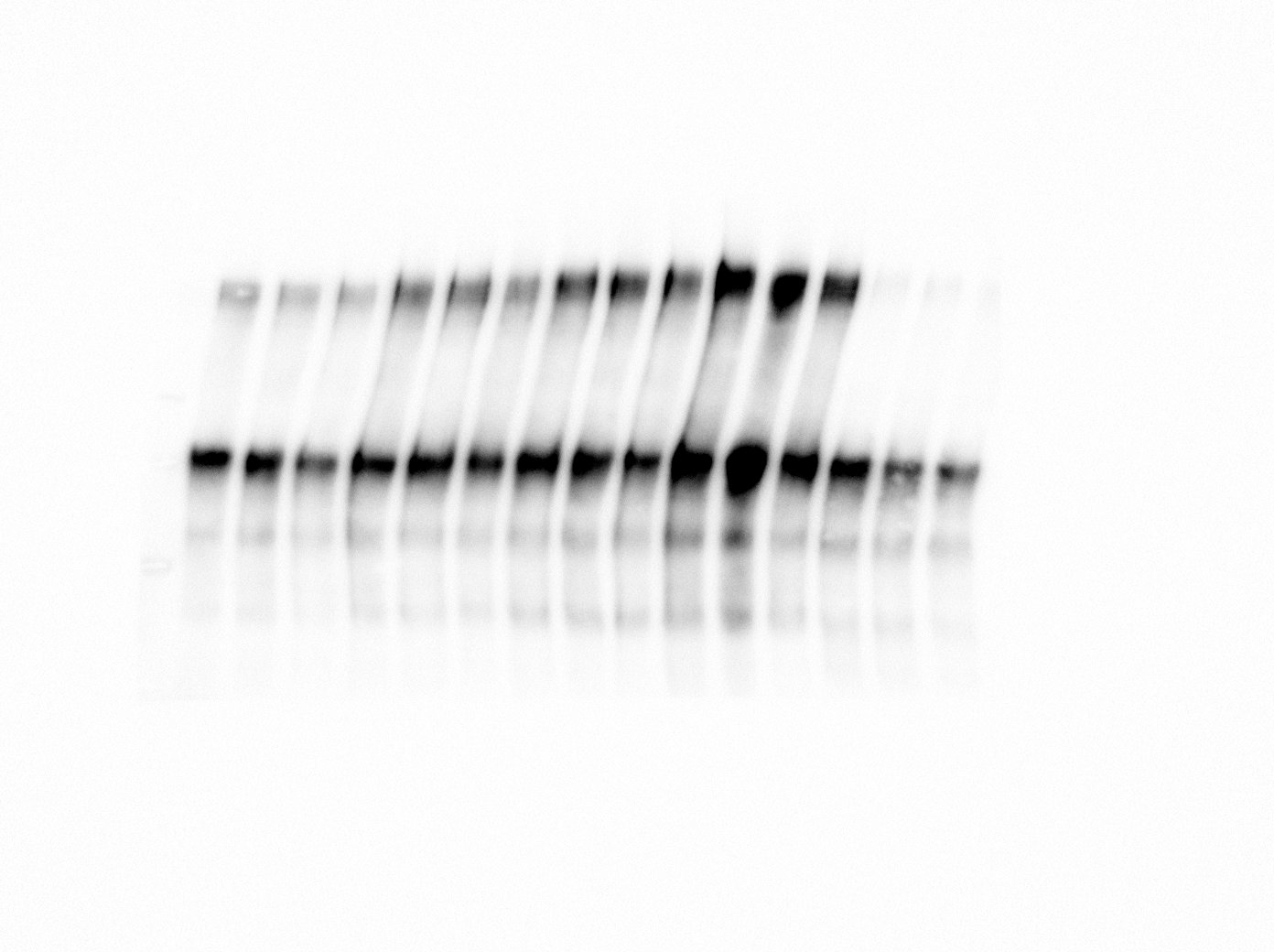

Supplement: Figure 1—source data 1. [file elife-92342-fig1-data1.zip › Figure 1-source data/Original blots for Fig1e/Fig1e NCAN (1).jpg]

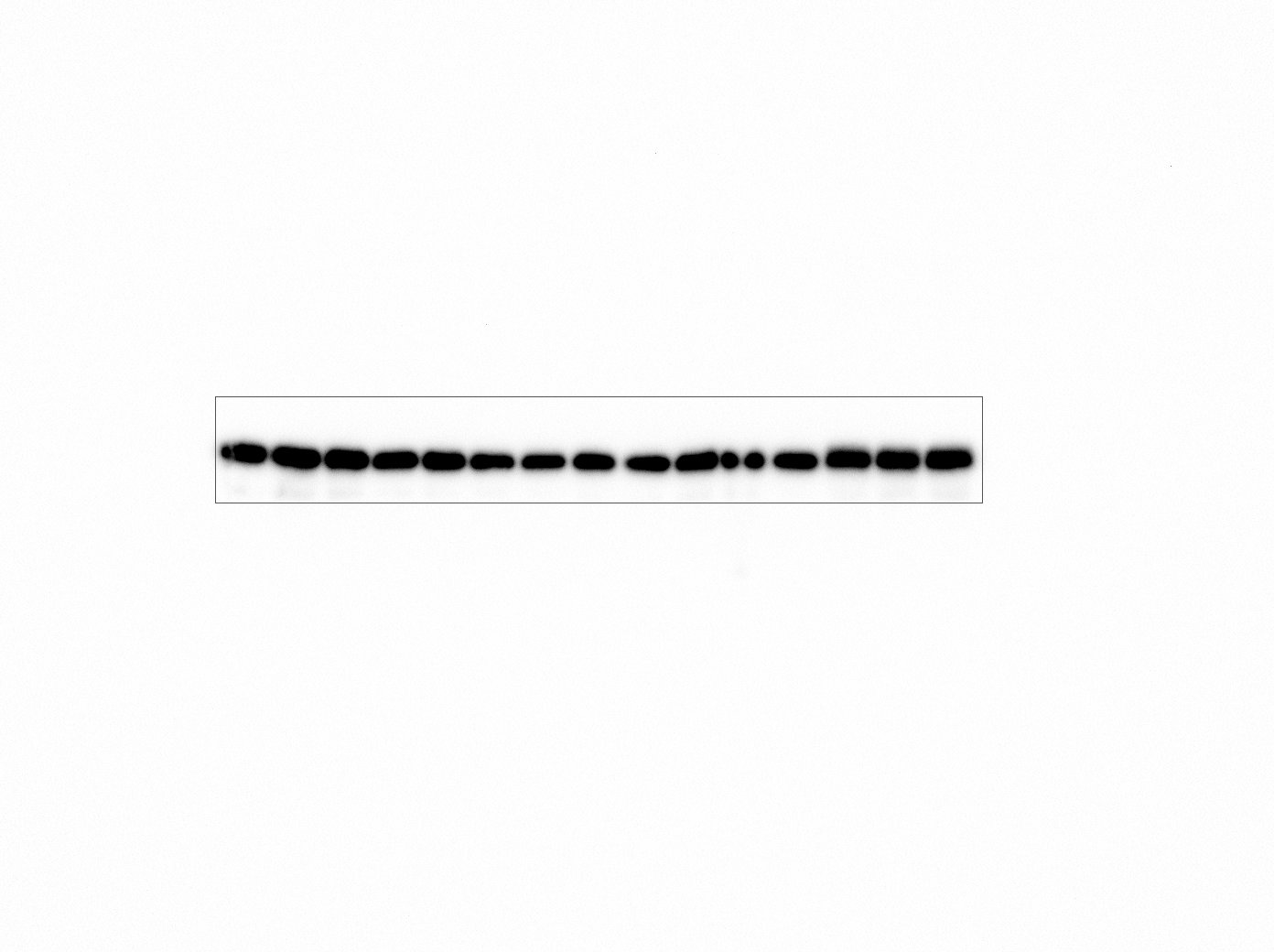

Supplement: Figure 1—source data 1. [file elife-92342-fig1-data1.zip › Figure 1-source data/Original blots for Fig1e/Fig1e GAPDH (2).jpg]

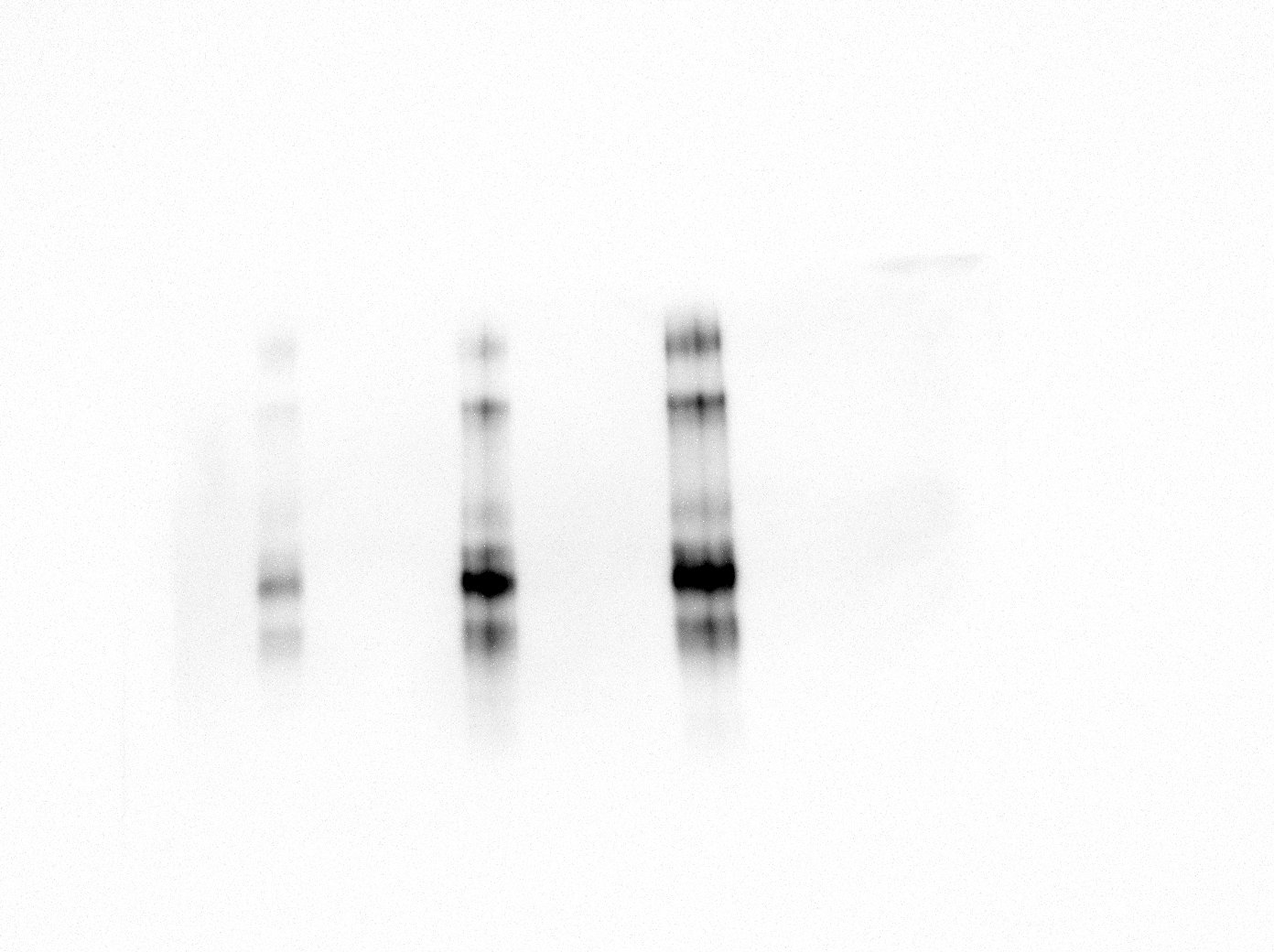

Supplement: Figure 1—source data 1. [file elife-92342-fig1-data1.zip › Figure 1-source data/Original blots for Fig1b/Fig1b (1).jpg]

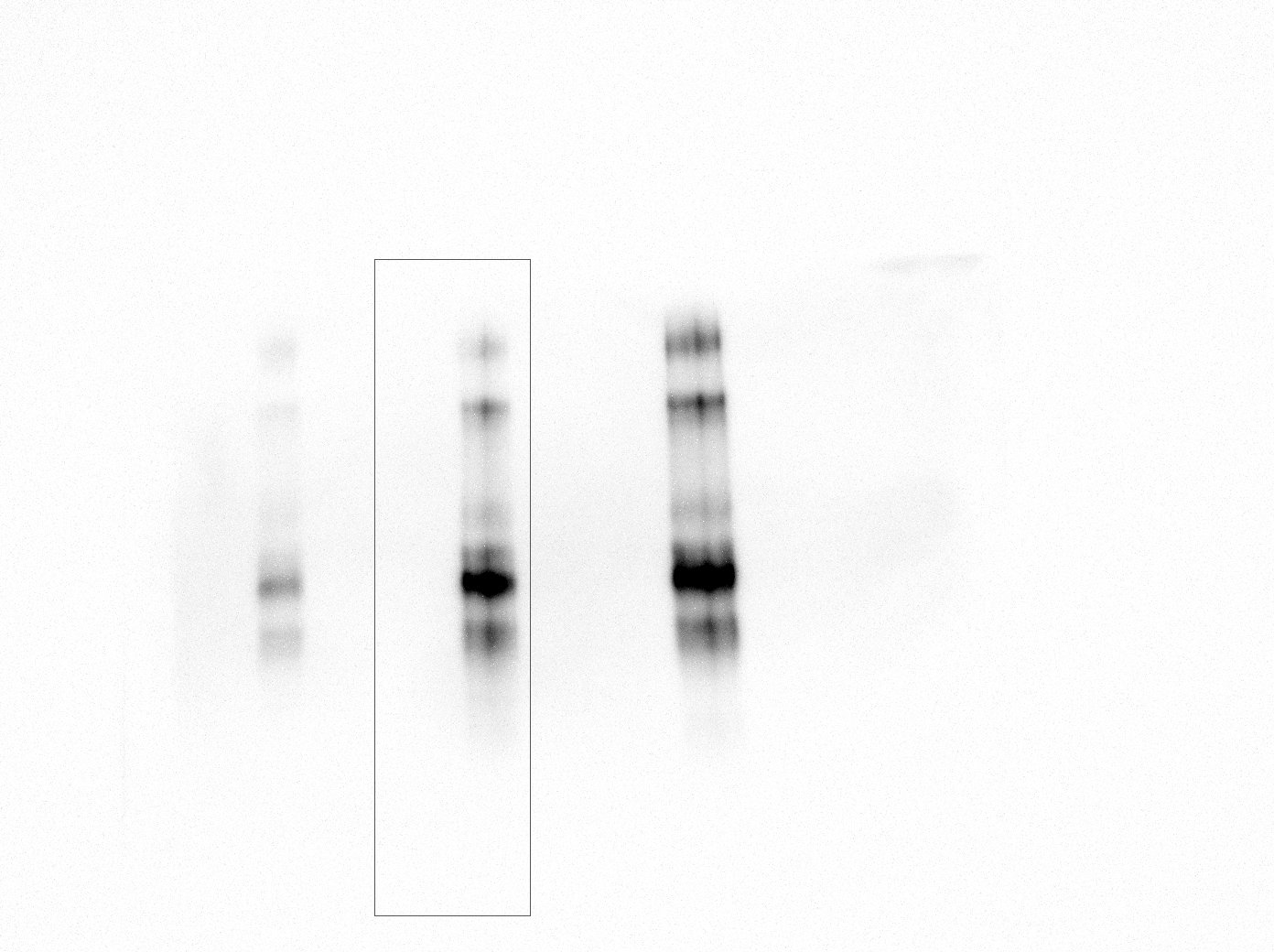

Supplement: Figure 1—source data 1. [file elife-92342-fig1-data1.zip › Figure 1-source data/Original blots for Fig1b/Fig1b (2).jpg]

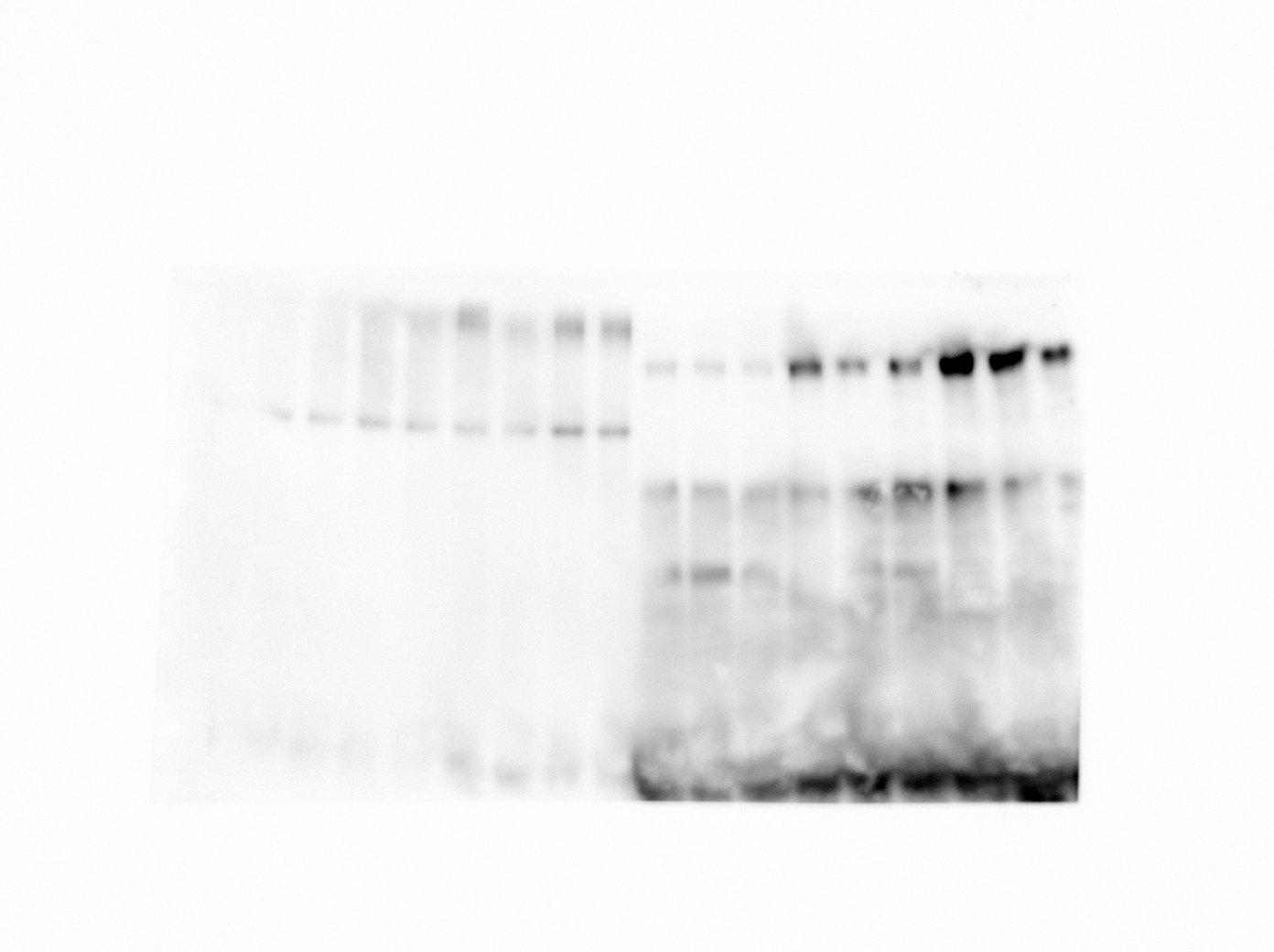

Supplement: Figure 1—source data 1. [file elife-92342-fig1-data1.zip › Figure 1-source data/Original blots for Fig1h/NCAN.jpg]

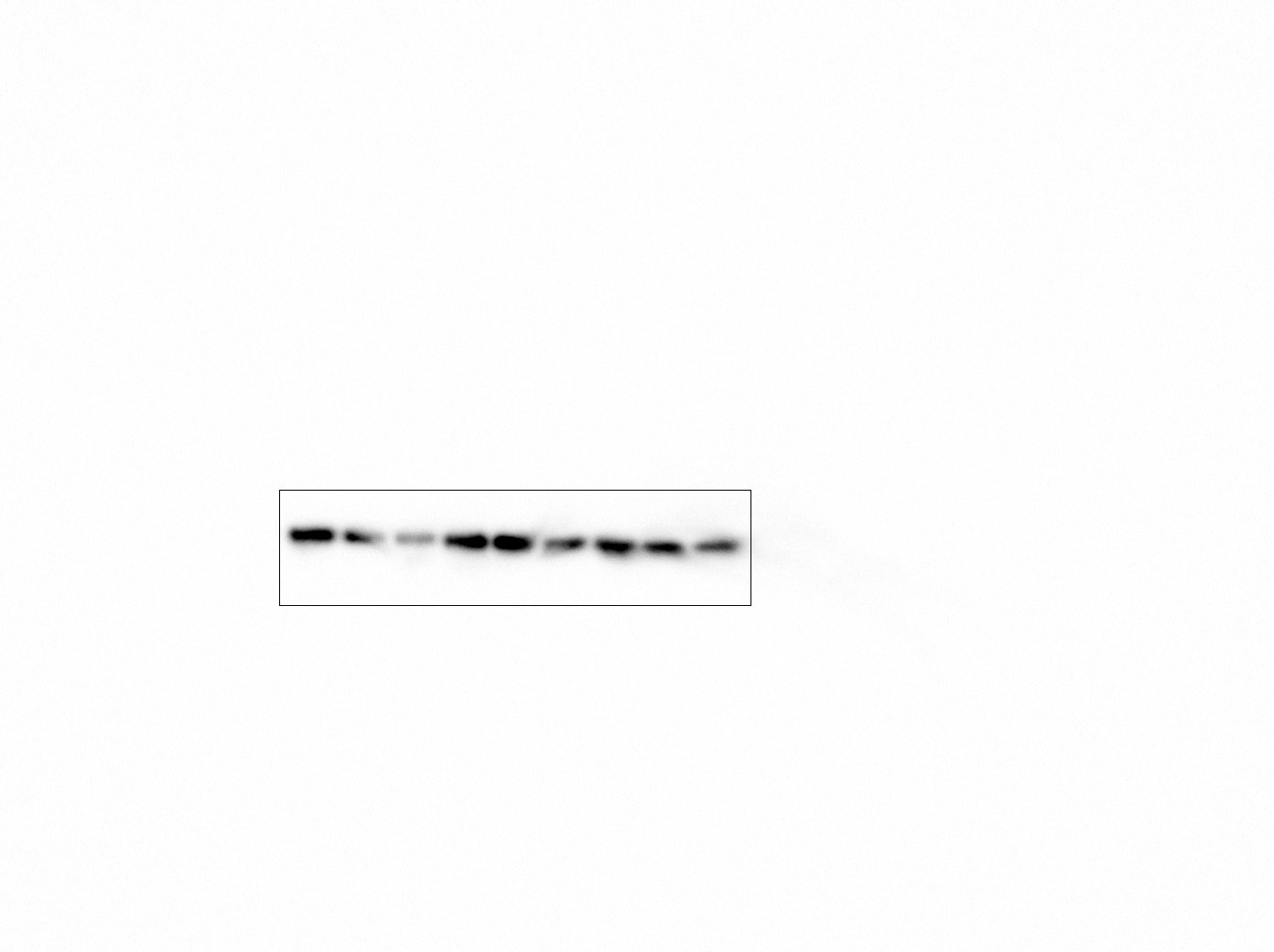

Supplement: Figure 1—source data 1. [file elife-92342-fig1-data1.zip › Figure 1-source data/Original blots for Fig1h/GAPDH_box.jpg]

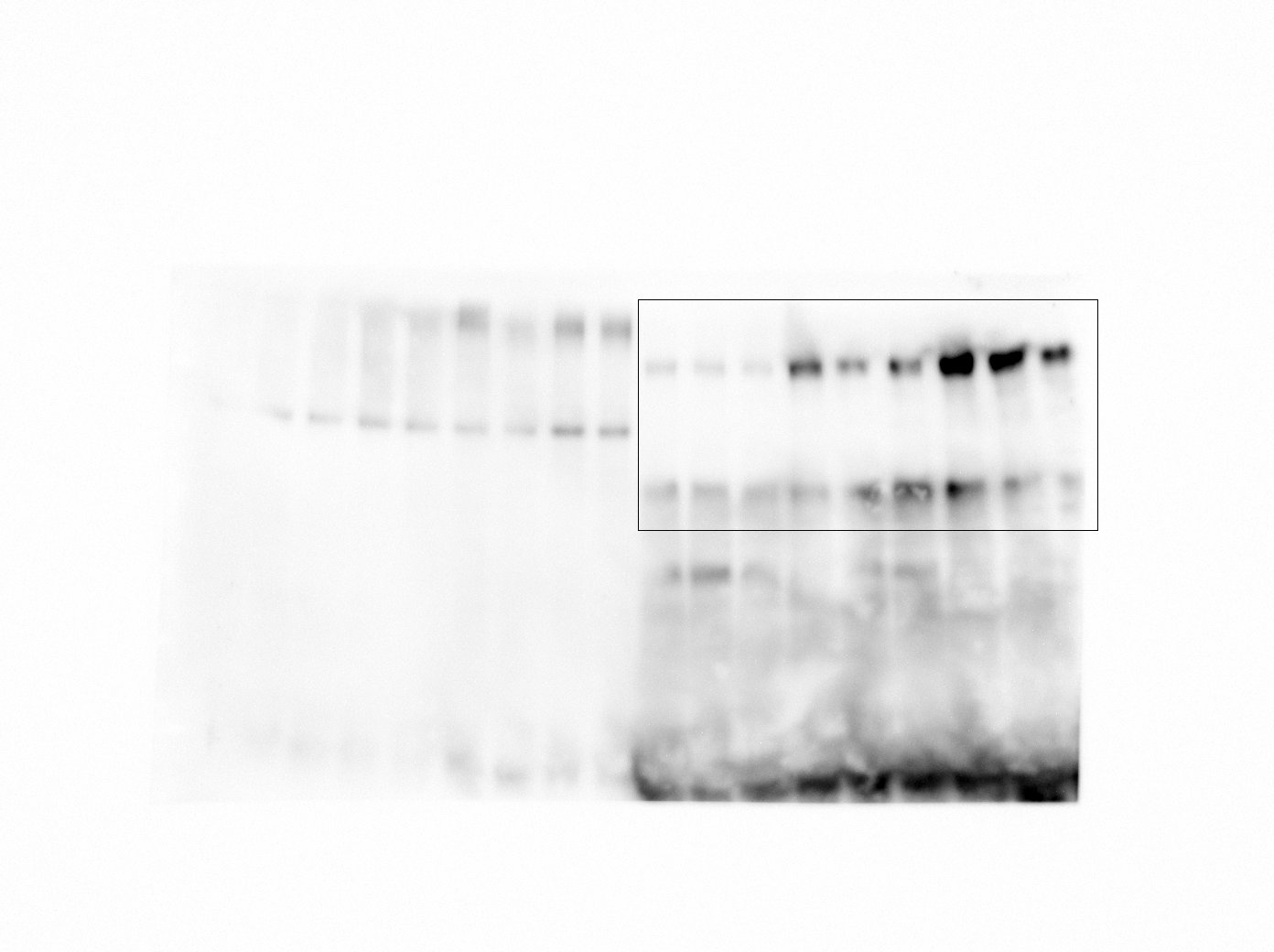

Supplement: Figure 1—source data 1. [file elife-92342-fig1-data1.zip › Figure 1-source data/Original blots for Fig1h/NCAN_box.jpg]

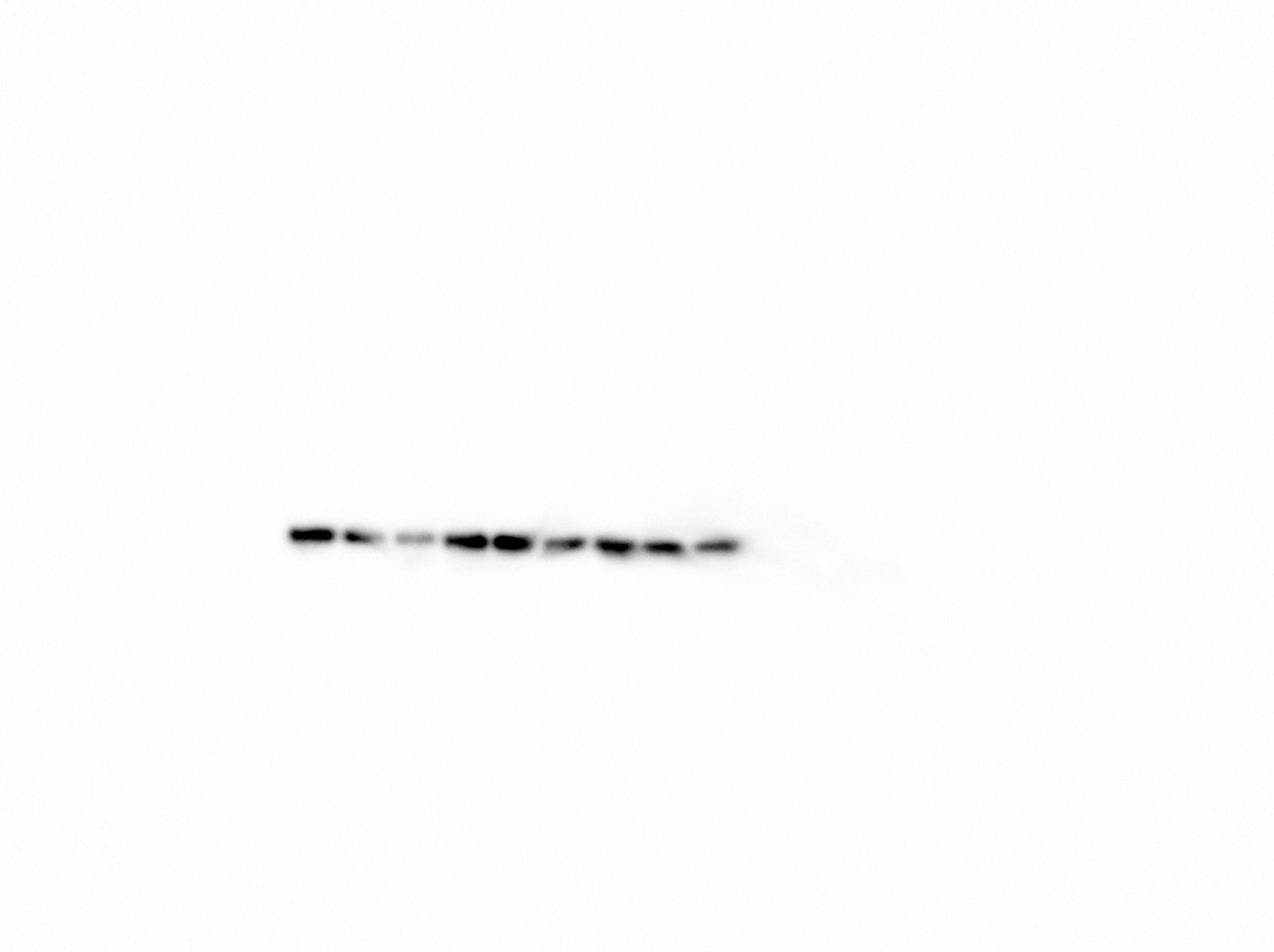

Supplement: Figure 1—source data 1. [file elife-92342-fig1-data1.zip › Figure 1-source data/Original blots for Fig1h/GAPDH.jpg]

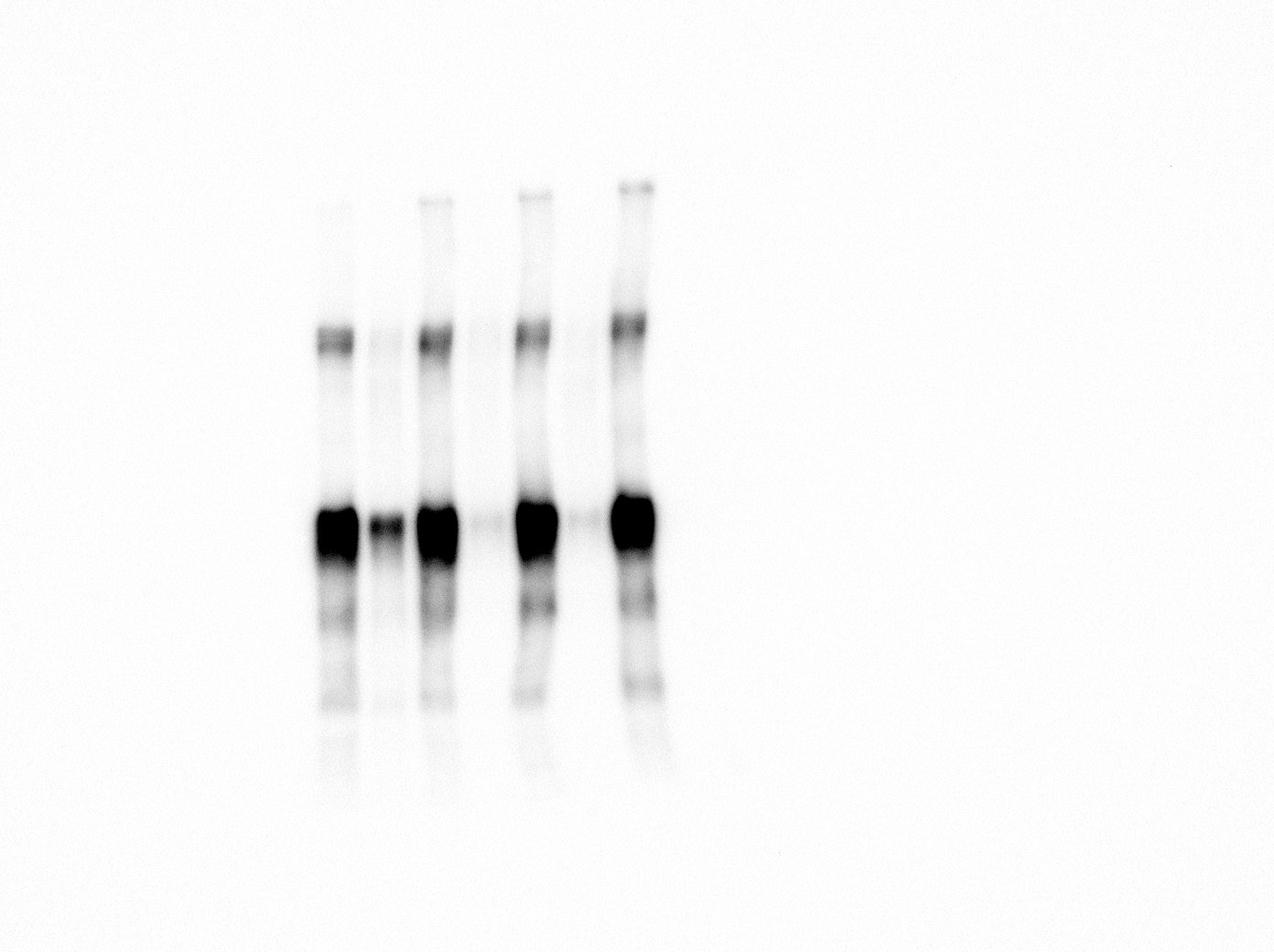

Supplement: Figure 2—source data 1. [file elife-92342-fig2-data1.zip › Figure 2-source data/Original blots for Fig2f/Fig2f (1).jpg]

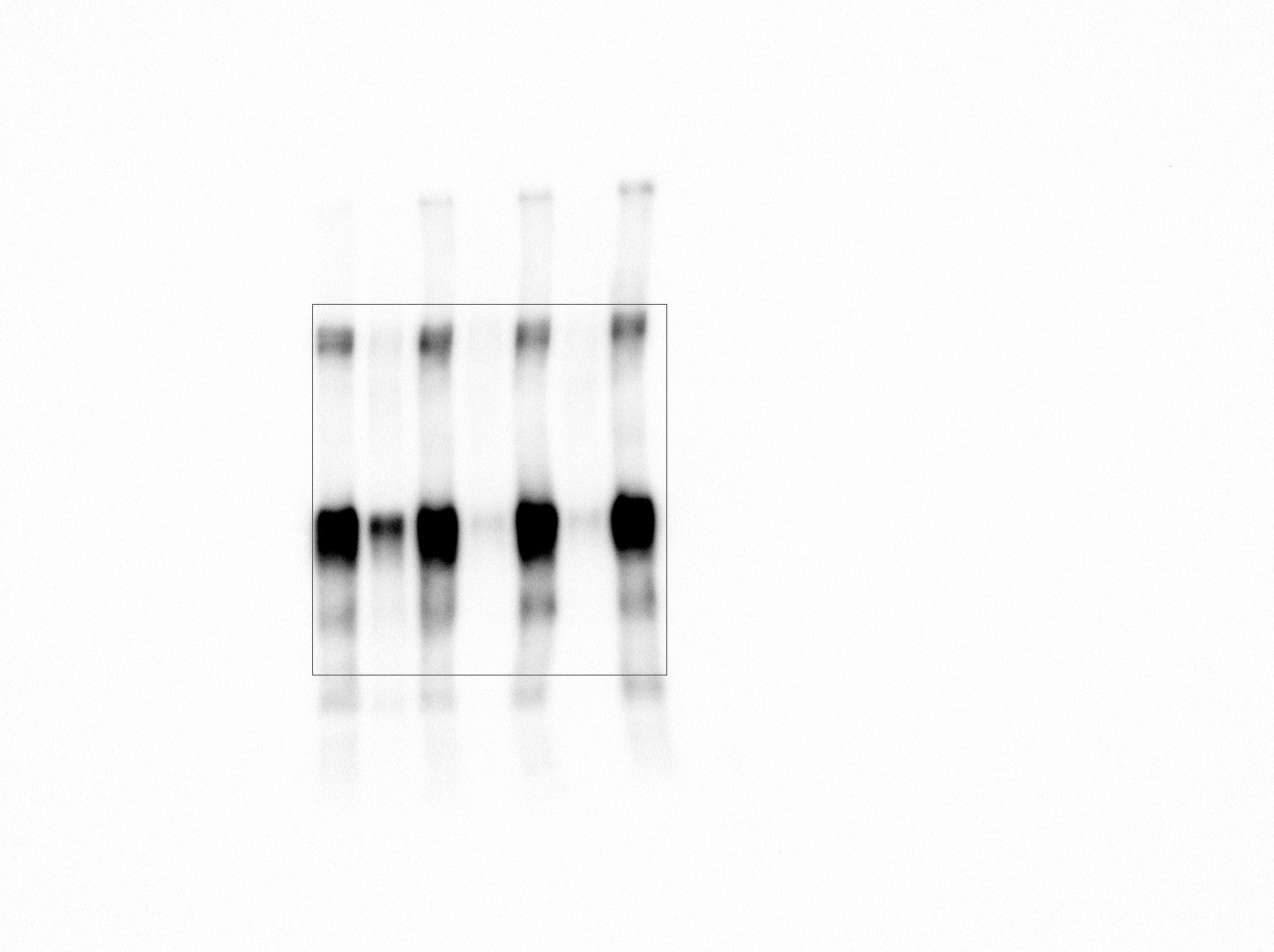

Supplement: Figure 2—source data 1. [file elife-92342-fig2-data1.zip › Figure 2-source data/Original blots for Fig2f/Fig2f (2).jpg]

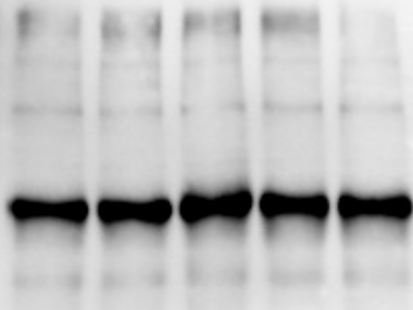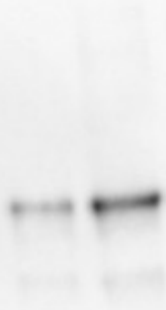

Supplement: Figure 2—source data 1. [file elife-92342-fig2-data1.zip › Figure 2-source data/Original blots for Fig2g/Fig2g (1).pdf]

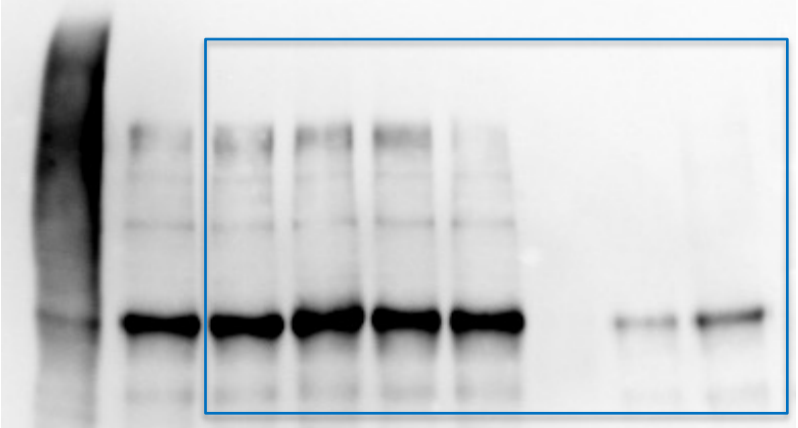

Supplement: Figure 2—source data 1. [file elife-92342-fig2-data1.zip › Figure 2-source data/Original blots for Fig2g/Fig2g (2).pdf]

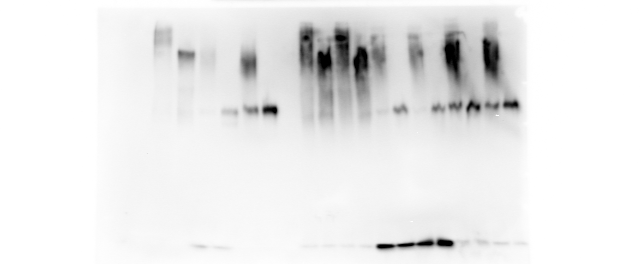

Supplement: Figure 2—figure supplement 1—source data 1. [file elife-92342-fig2-figsupp1-data1.zip › Figure 2-figure supplement 1-source data/Original blots for Figure 2-figure supplement 1/Fig2 Sup1a (1).tif]

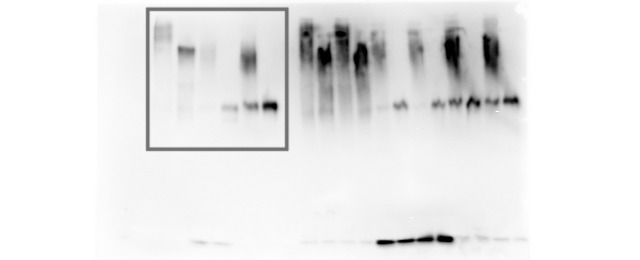

Supplement: Figure 2—figure supplement 1—source data 1. [file elife-92342-fig2-figsupp1-data1.zip › Figure 2-figure supplement 1-source data/Original blots for Figure 2-figure supplement 1/Fig2 Sup1a (2).tif]

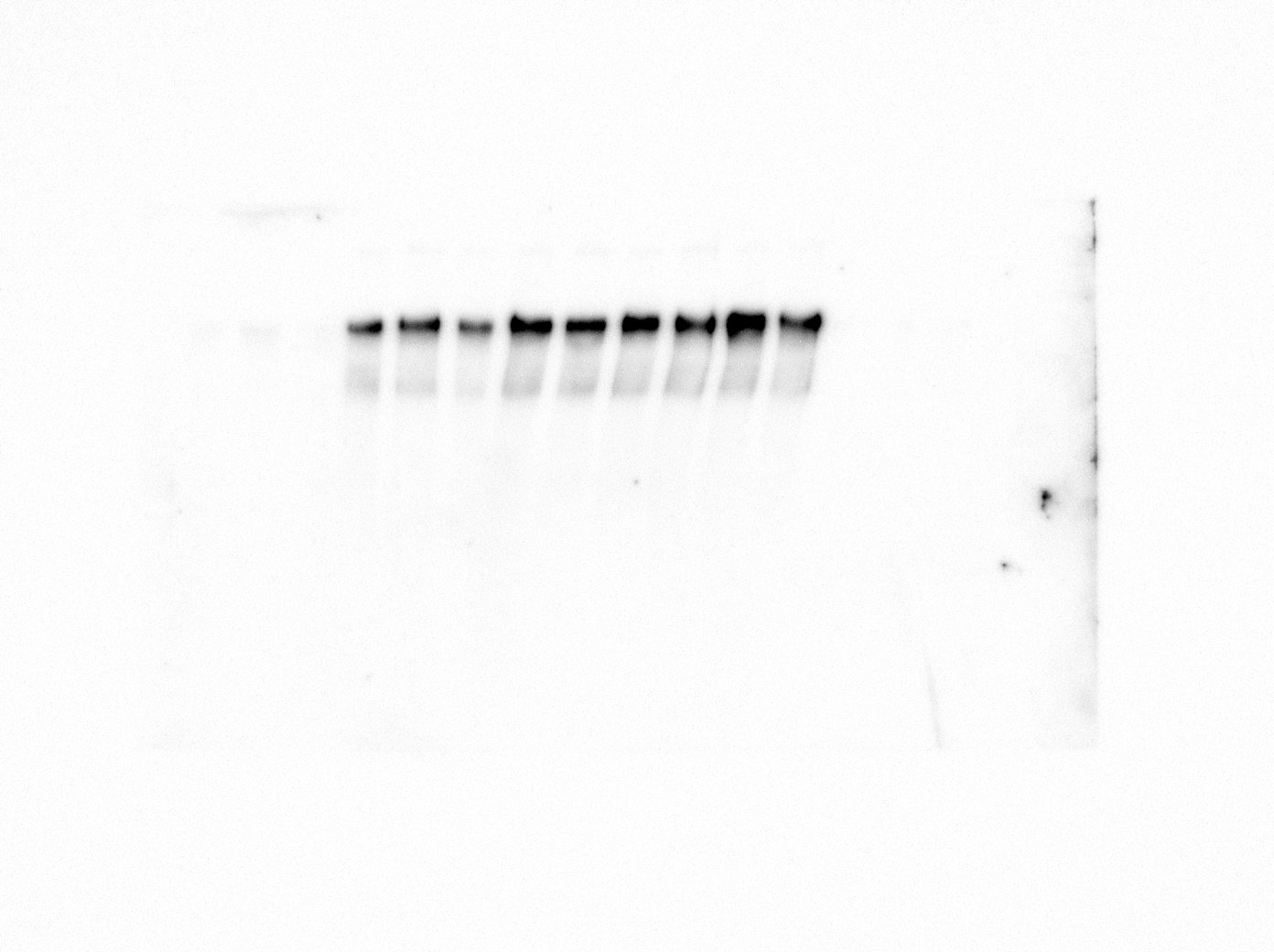

Supplement: Figure 3—source data 1. [file elife-92342-fig3-data1.zip › Figure 3-source data/Original blots for Fig3c/Fig3 c (1).jpg]

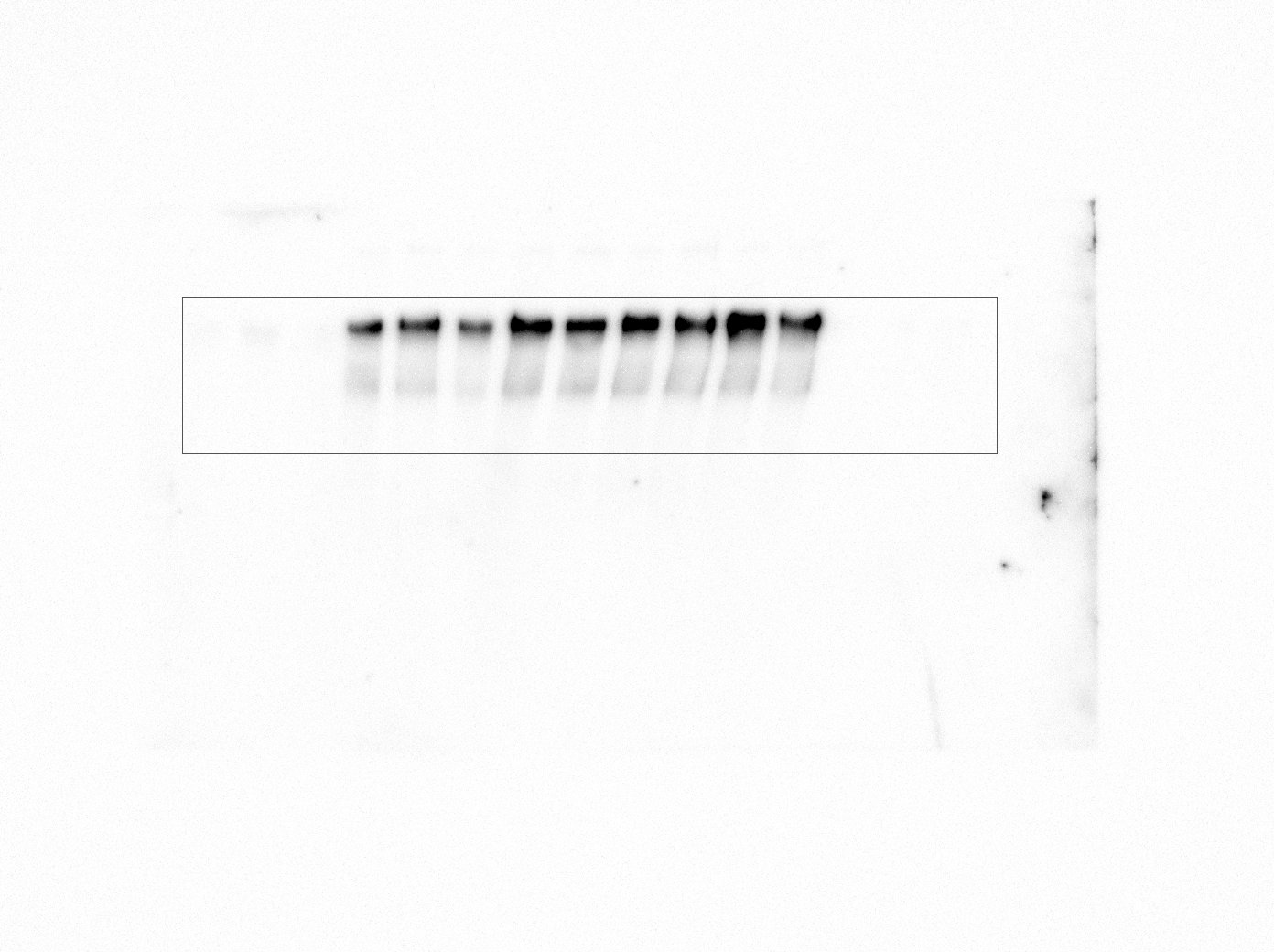

Supplement: Figure 3—source data 1. [file elife-92342-fig3-data1.zip › Figure 3-source data/Original blots for Fig3c/Fig3 c (2).jpg]

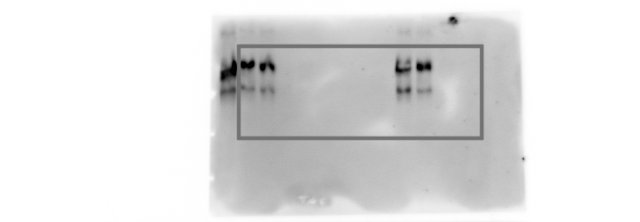

Supplement: Figure 3—source data 1. [file elife-92342-fig3-data1.zip › Figure 3-source data/Original blots for Fig3b/Fig3b (2).tif]

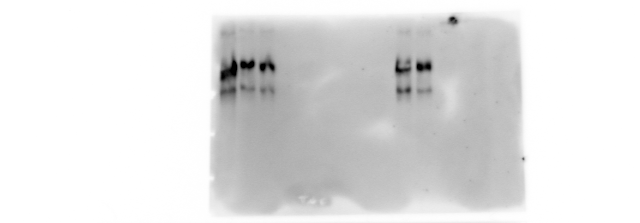

Supplement: Figure 3—source data 1. [file elife-92342-fig3-data1.zip › Figure 3-source data/Original blots for Fig3b/Fig3b (1).tif]

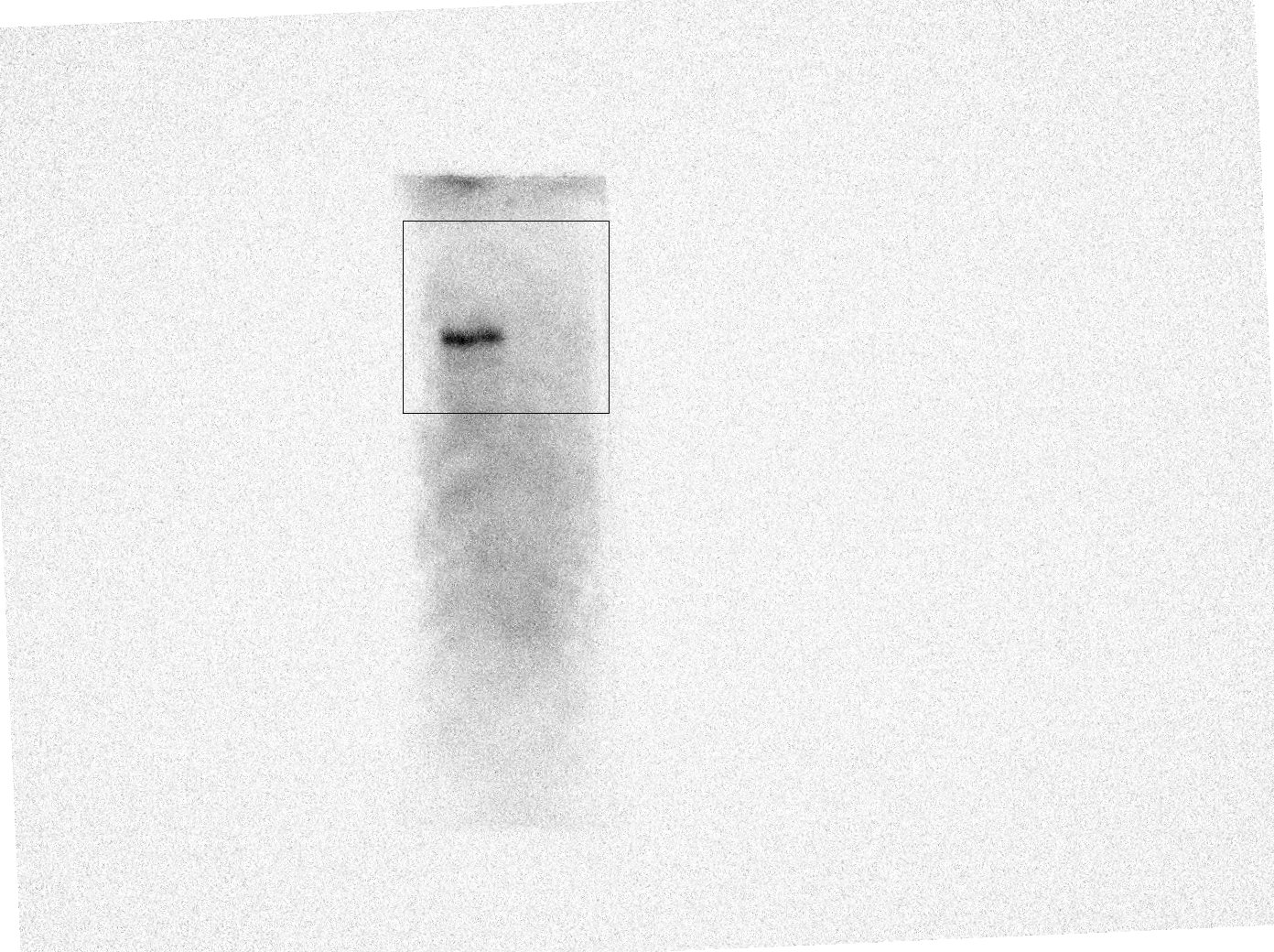

Supplement: Figure 6—figure supplement 1—source data 1. [file elife-92342-fig6-figsupp1-data1.zip › Figure 6-figure supplement 1-source data/Original blots for Figure 6-figure supplement 1/Fig6 Sup1a TNC.jpg]

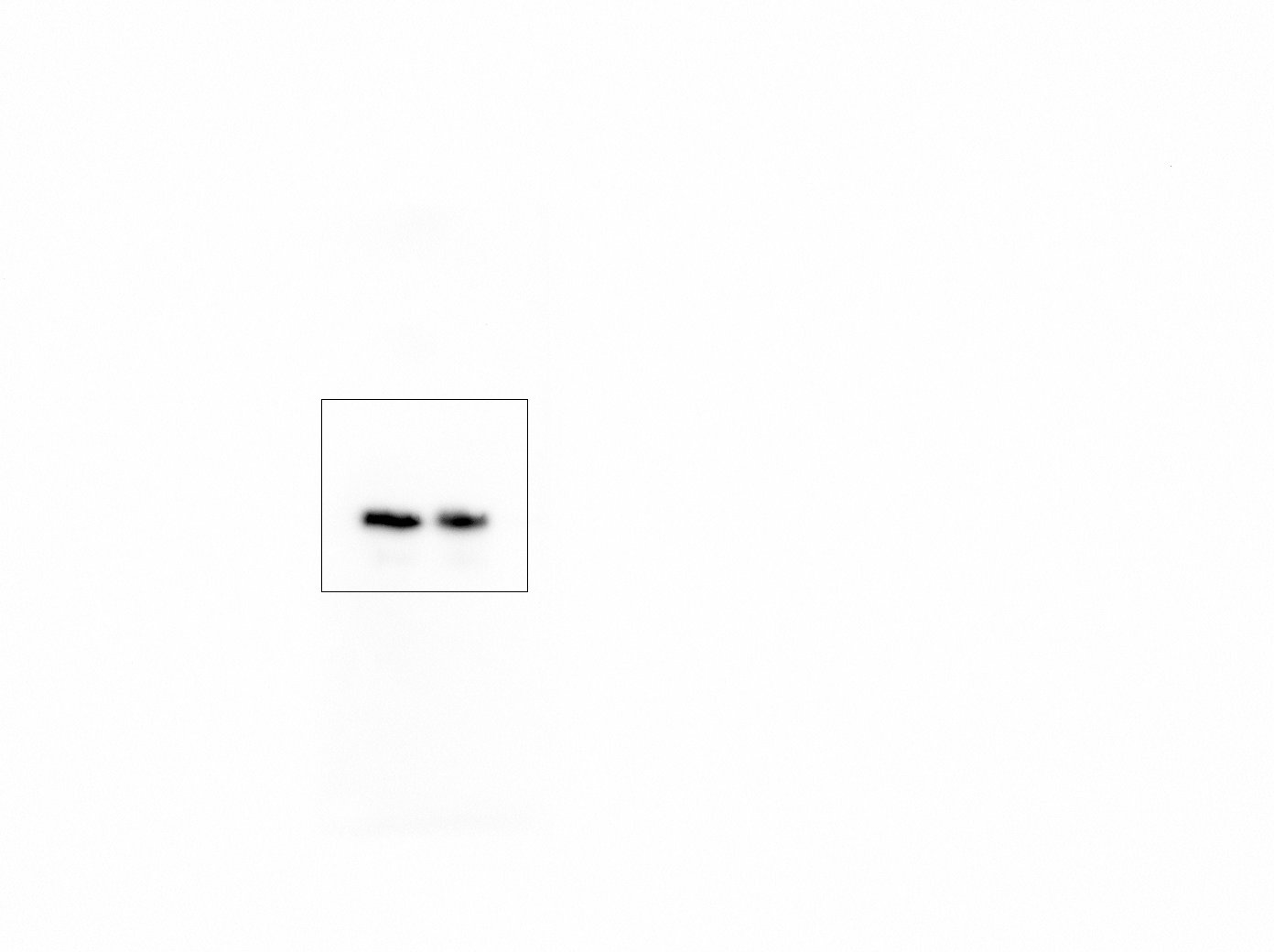

Supplement: Figure 6—figure supplement 1—source data 1. [file elife-92342-fig6-figsupp1-data1.zip › Figure 6-figure supplement 1-source data/Original blots for Figure 6-figure supplement 1/Fig6 Sup1a GAPDH.jpg]

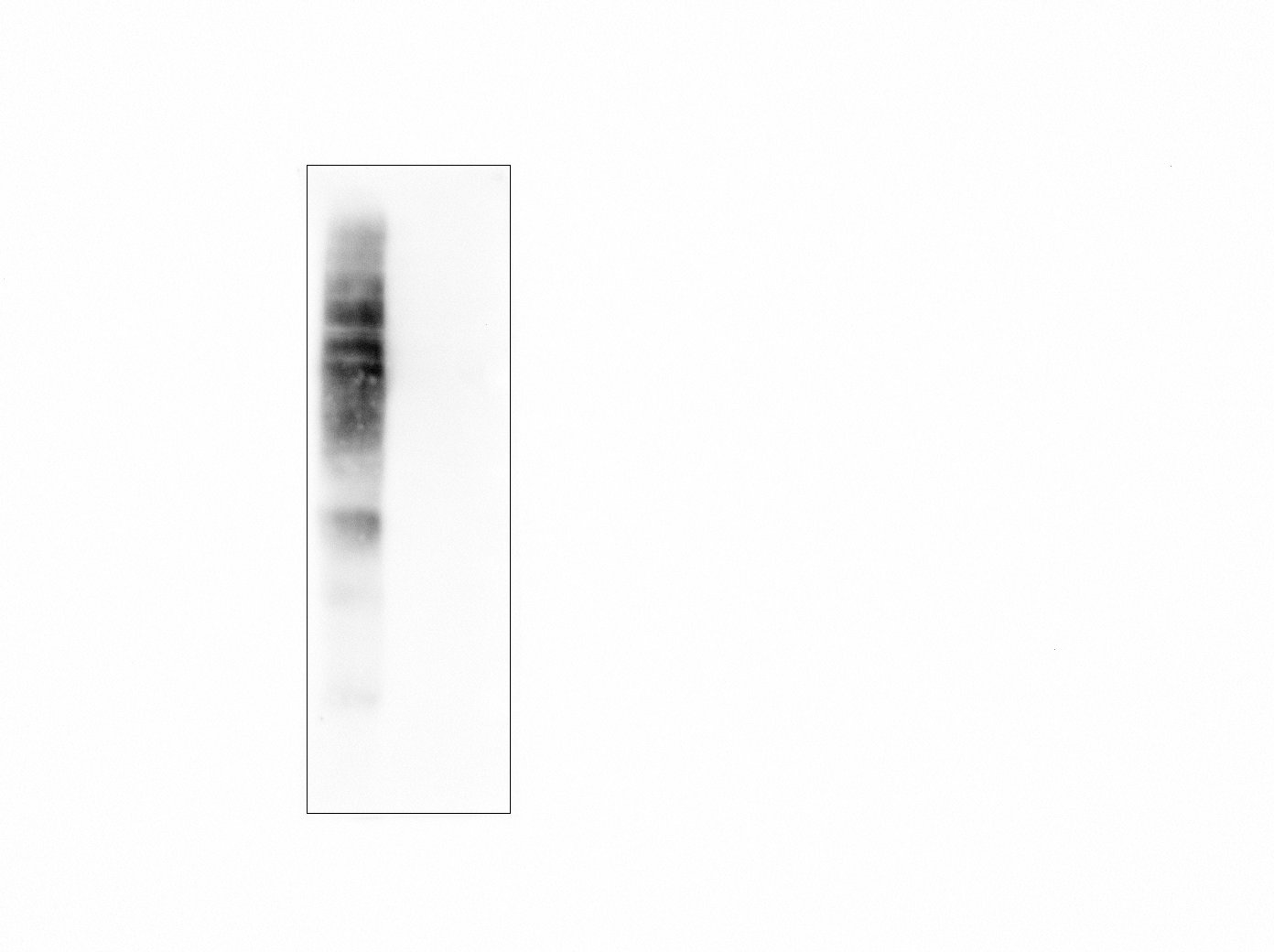

Supplement: Figure 6—figure supplement 1—source data 1. [file elife-92342-fig6-figsupp1-data1.zip › Figure 6-figure supplement 1-source data/Original blots for Figure 6-figure supplement 1/Fig6 Sup1a NCAN.jpg]

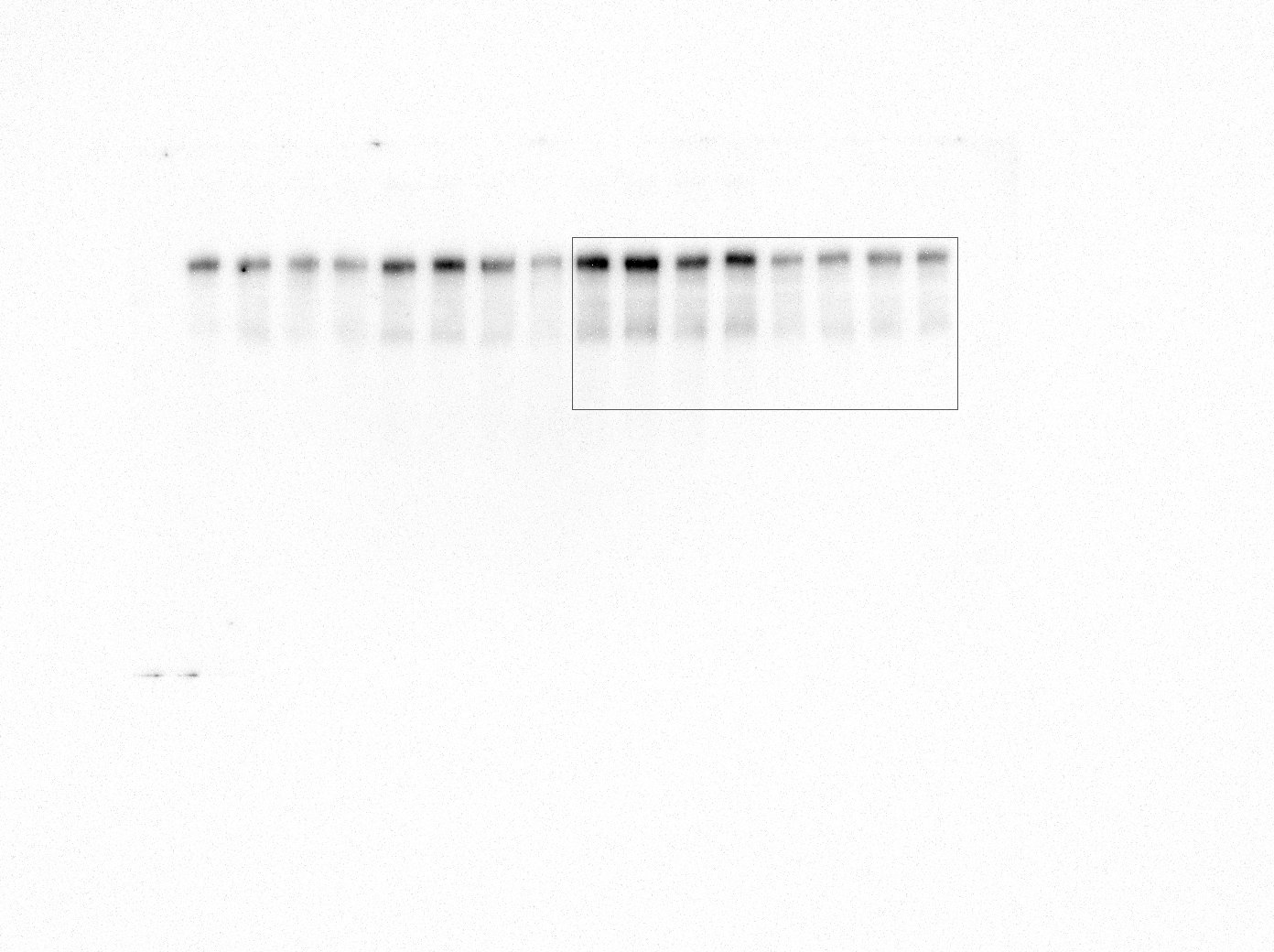

Supplement: Figure 8—source data 1. [file elife-92342-fig8-data1.zip › Figure 8-source data/Original blots for Fig8b/Fig8 b TNC (2).jpg]

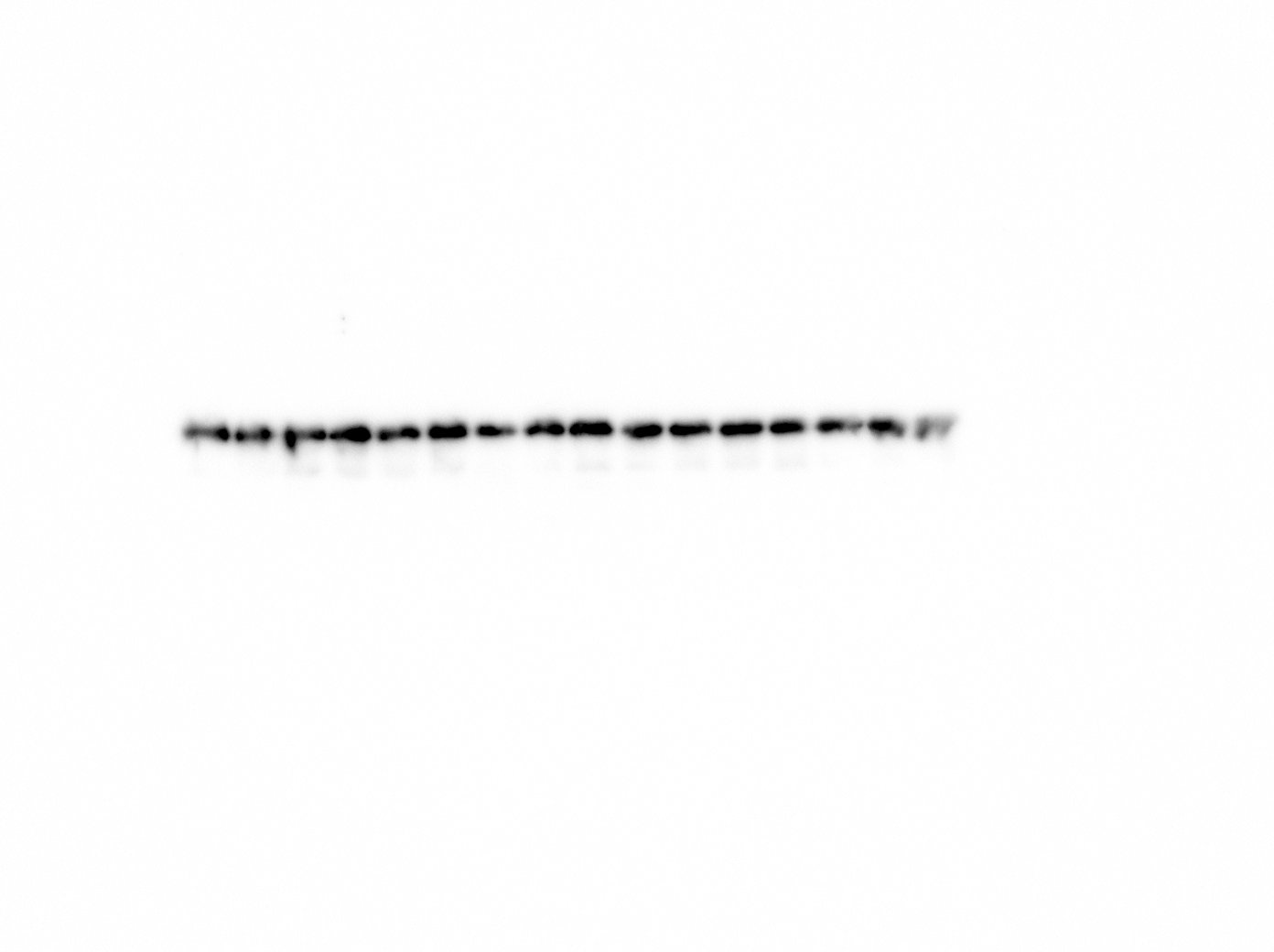

Supplement: Figure 8—source data 1. [file elife-92342-fig8-data1.zip › Figure 8-source data/Original blots for Fig8b/Fig8 b GAPDH (1).jpg]

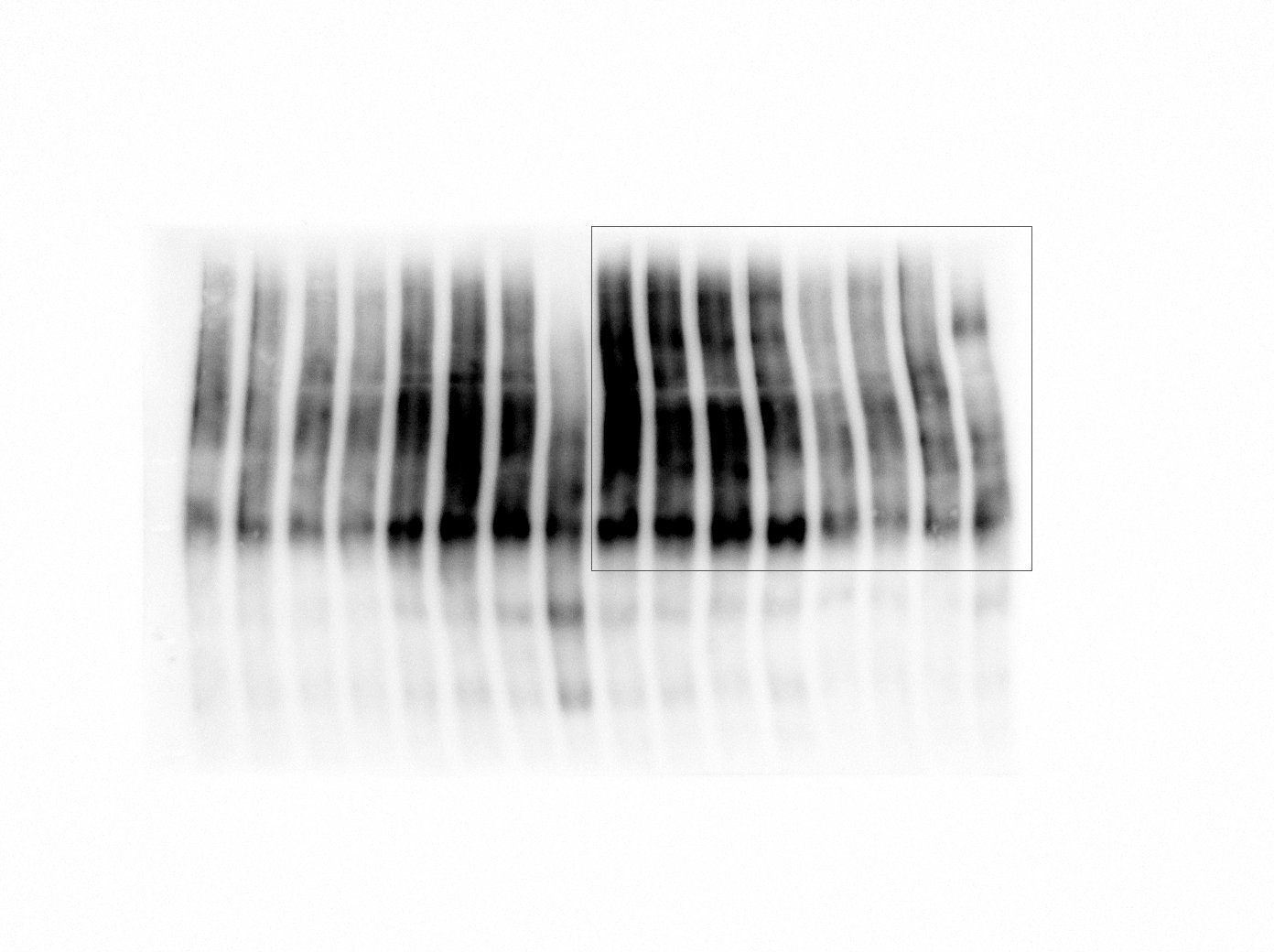

Supplement: Figure 8—source data 1. [file elife-92342-fig8-data1.zip › Figure 8-source data/Original blots for Fig8b/Fig8 b NCAN (2).jpg]

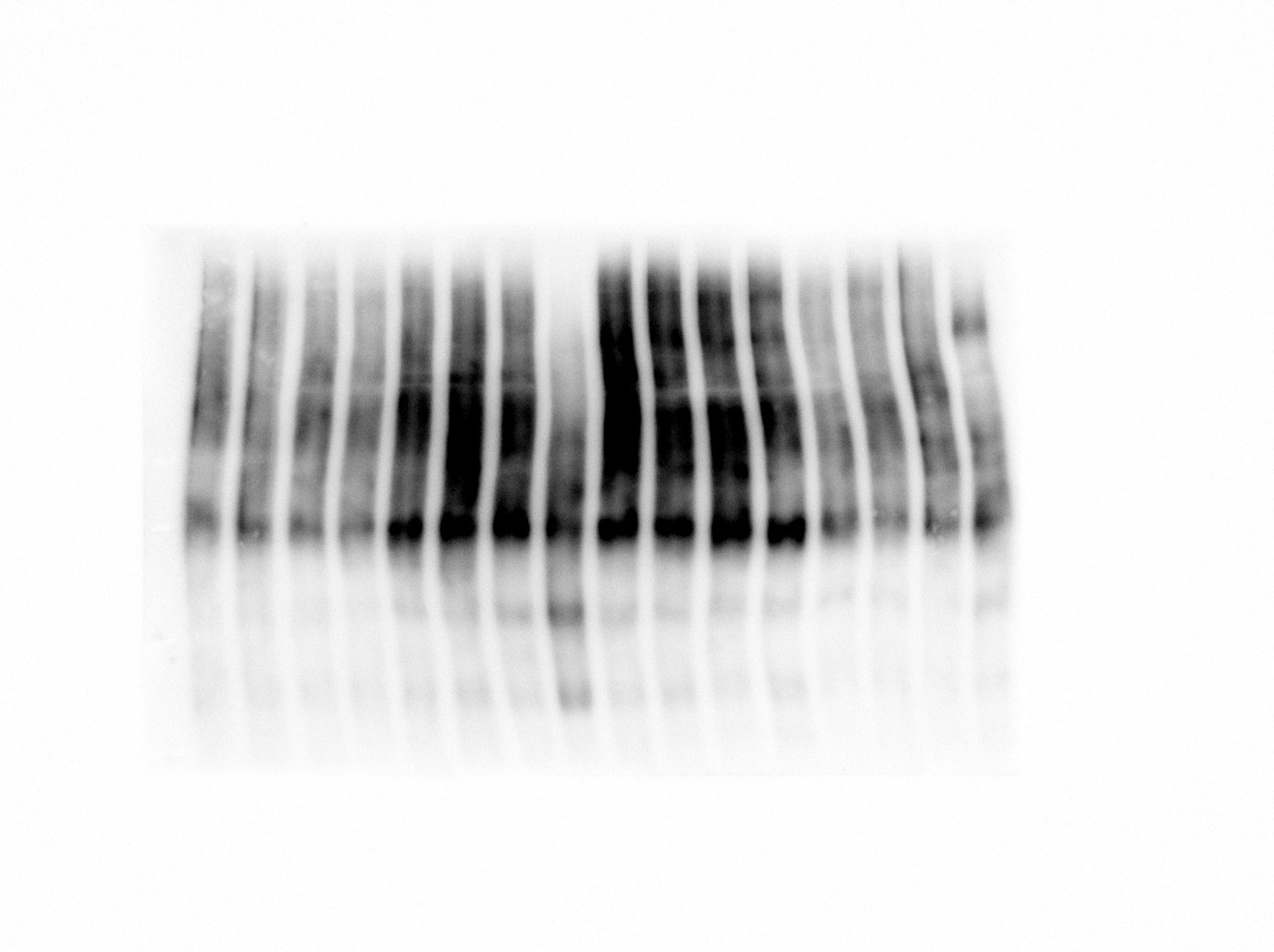

Supplement: Figure 8—source data 1. [file elife-92342-fig8-data1.zip › Figure 8-source data/Original blots for Fig8b/Fig8 b NCAN (1).jpg]

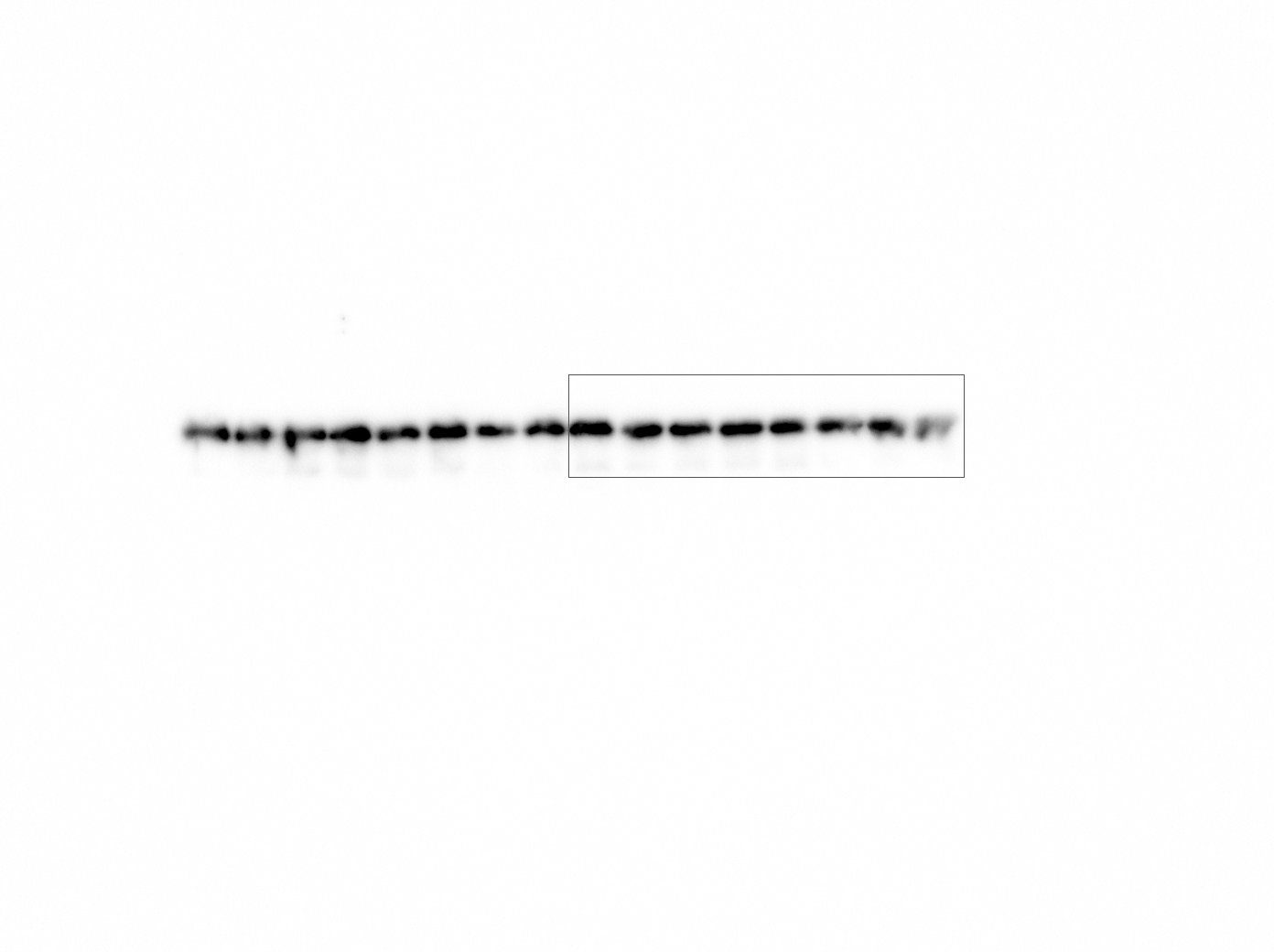

Supplement: Figure 8—source data 1. [file elife-92342-fig8-data1.zip › Figure 8-source data/Original blots for Fig8b/Fig8 b GAPDH (2).jpg]

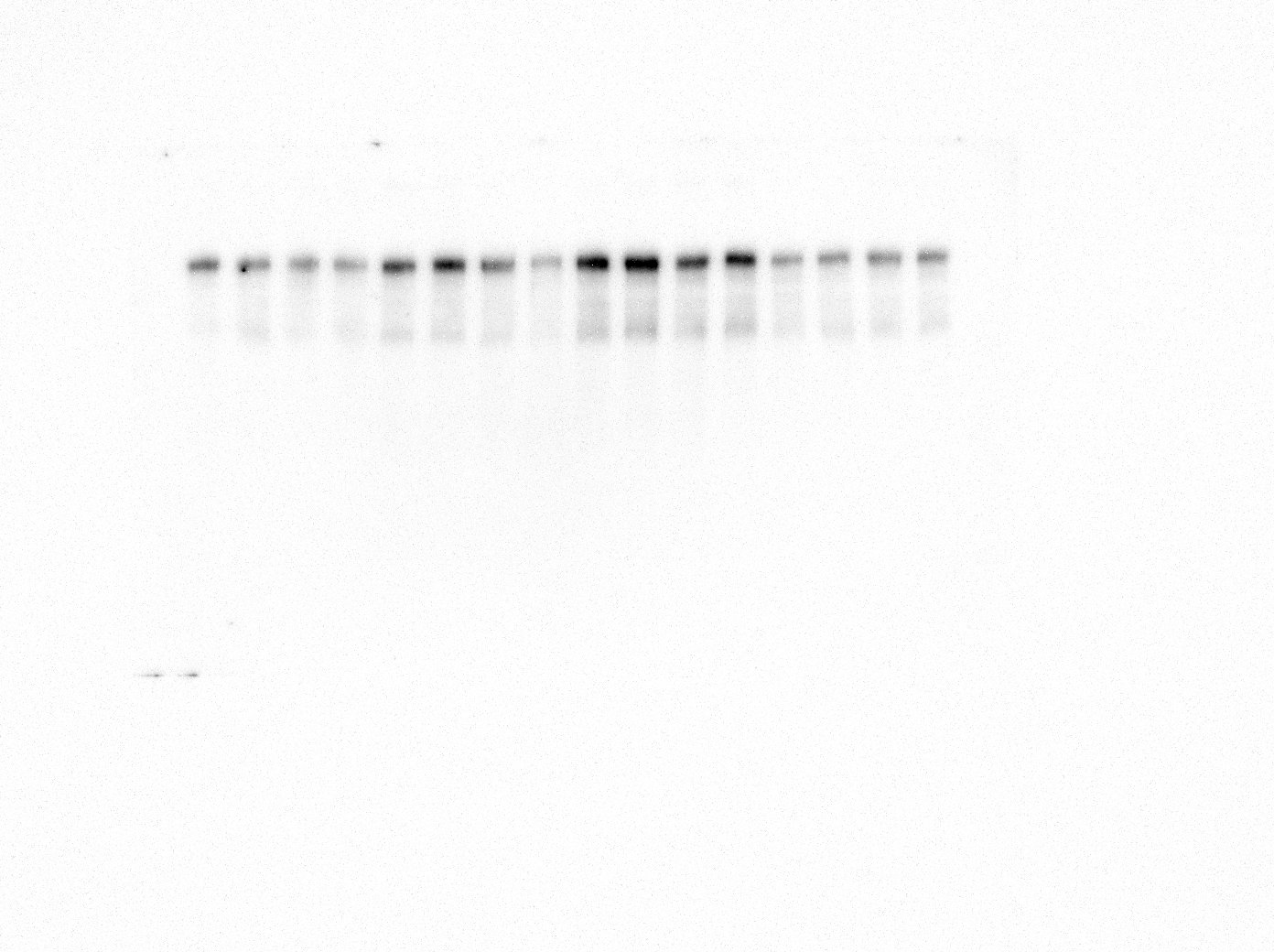

Supplement: Figure 8—source data 1. [file elife-92342-fig8-data1.zip › Figure 8-source data/Original blots for Fig8b/Fig8 b TNC (1).jpg]
